# A multicohort machine-learning prognostic signature reveals inflammatory immune remodeling and statin sensitivity in glioblastoma

**DOI:** 10.3389/fonc.2026.1912201

**Published:** 2026-08-05

**Authors:** Zhe Xing, Han Yang, Yuan Lyu, Junqi Li, Xueli Tian, Qiao Shan, Wulong Liang, Weihua Hu, Shaolong Zhou, Xinjun Wang

**Affiliations:** 1 Department of Neurosurgery, The Fifth Affiliated Hospital of Zhengzhou University, Zhengzhou, Henan, China; 2 Henan International Joint Laboratory of Glioma Metabolism and Microenvironment Research, Zhengzhou, Henan, China; 3 Institute of Neuroscience, Zhengzhou University, Zhengzhou, Henan, China; 4 Department of Clinical Research and Translational Medicine, The Third Affiliated Hospital of Zhengzhou University, Zhengzhou, Henan, China; 5 Tianjian Laboratory of Advanced Biomedical Sciences, School of Life Sciences, Zhengzhou University, Zhengzhou, Henan, China; 6 Institute of Development and Metabolism, Zhengzhou University, Zhengzhou, Henan, China; 7 Henan Pediatric Brain Glioma Precision Diagnosis and Treatment Engineering Research Center, Zhengzhou, Henan, China; 8 Department of Neurosurgery, The Third Affiliated Hospital of Zhengzhou University, Zhengzhou, Henan, China

**Keywords:** biomarker, computational biology and bioinformatics, drug discovery, glioblastoma, immune landscape

## Abstract

**Introduction:**

Glioblastoma (GBM) is characterized by substantial inter- and intra-tumoral heterogeneity. A robust multigene model may help address this heterogeneity and improve prognostic stratification.

**Methods:**

We integrated 76 combinations derived from 10 machine-learning algorithms to develop a purificatory machine learning-derived gene signature (PMLS). The model was evaluated in 10 public cohorts comprising 1,097 patients and compared with common clinicopathological variables and 135 published prognostic signatures. Biological pathways, immune characteristics, and potential therapeutic agents associated with PMLS were further investigated.

**Results:**

PMLS independently predicted patient prognosis and demonstrated robust performance across all cohorts. It outperformed common clinical and molecular characteristics and most published signatures. Higher PMLS scores were associated with increased proliferation, inflammatory signaling, altered molecular features, and extensive immune-microenvironment remodeling. Simvastatin and fluvastatin were identified as potential therapeutic candidates for high-risk patients.

**Discussion:**

PMLS may provide a robust platform for prognostic stratification, biological interpretation, and the development of precision-treatment strategies for patients with GBM.

## Background

Gliomas are the most common and most lethal type of primary brain tumors, originating from glial cells (1). The latest version of WHO CNS5 in 2021 was founded on the histological features of gliomas from grade I to IV in the WHO categorization in 2016, with the addition of updated molecular biomarkers for distinct tumor types, enabling patients to receive meaningful guidance and yield more benefits (2, 3). Gliomas are bifurcated into benign circumscribed gliomas and highly malignant diffuse gliomas with the first being curable through complete resection while the latter cannot be cured through surgical resection alone. Likewise, the grading of cancer types is also of great significance, with the following 4 families: (1) circumscribed astrocytic gliomas; (2) adult-type diffuse gliomas; (3) pediatric-type diffuse high-grade gliomas; (4) pediatric-type diffuse low-grade gliomas (4). Generally, according to the clinicopathological grading, CNS WHO grades 1–2 are categorized as low-grade gliomas (LGG), while grades 3–4 are categorized as high-grade gliomas (HGG). LGGs present a relatively promising prognosis, whereas they account for only a small proportion (6% of CNS primary tumors), and some inevitably recur and progress to HGGs (5). Glioblastoma (GBM) accounts for a large proportion (48% of primary brain tumors and 57% of all gliomas) and is one of the deadliest and most inclined to relapse malignant tumors, with a median survival time shorter than 2 years and an overall survival (OS) rate at 5 years of less than 5% (6, 7). Maximal surgical removal followed by concomitant radiotherapy and temozolomide (TMZ) chemotherapy constitutes the prevailing standard of care for newly diagnosed GBM patients (8). Despite optimal treatment and the flourishing of new pharmaceuticals, no other therapeutic interventions have been found to prolong OS except for tumor-treating fields, and all GBMs eventually progress (9). GBMs can be diagnosed by standard magnetic resonance imaging (MRI), but it has commonly developed into advanced stages at the time of diagnosis (10). Thus, it is urgent to identify reliable biomarkers for early patient stratification and to ascertain the benefits of drug discovery.

GBM is typified by both inter- and intratumor heterogeneity. An ideal biomarker should sustain homogenous expression within and across tumors and be robust for all patients. Consequently, a multigene panel could be a favorable strategy to solve this problem. Along with the unceasing progress of bioinformatics technology, a profusion of prognostic gene signatures integrated by multigene profiles, such as microRNAs (miRNAs), messenger RNAs (mRNAs), and long noncoding RNAs (lncRNAs), have been developed for GBM (11–13). However, these signatures are rarely applied in clinical settings due to underdeveloped data information, unreasonable machine learning implementation, and a lack of stringent validation by different datasets. Therefore, prognostic signatures require rigorous development and validation before clinical translation.

In our study, we endeavored to comprehensively integrate genomic and epigenetic information and methodically developed a purificatory machine learning-derived gene signature (PMLS) through 76 kinds of algorithm combinations based on 10 machine learning methods in GBM. A total of 1097 patients from 10 independent cohorts were exploited to identify and verify the PMLS regarding OS, available clinical traits and molecular features. The C-indices of the PMLS and 135 published signatures were computed and compared across all cohorts to demonstrate the prior prediction performance of our model. We also focused on investigating the potential biological mechanisms underlying PMLS and explored prospective drugs for treating GBM patients with high PMLS scores.

## Materials and methods

### Data acquisition from public platforms

To maximize the applicability and robustness of the consensus model, we winnowed multiple types (sequencing and array) of expression data from multiple databases annotated on multiple platforms. The general flow of our study is displayed in **Supplementary Figure 1**. A total of 1630 samples from 10 independent GBM cohorts were initially retrieved from public databases. One was from The Cancer Genome Atlas (TCGA), named TCGA-GBM; two were from the Chinese Glioma Genome Atlas (CGGA), including mRNA-array_301 and mRNAseq_325 (named c301 and c325 respectively); and seven were from Gene Expression Omnibus (GEO), including GSE13041, GSE42669, GSE43378, GSE74187, GSE83300, GSE4271, and GSE4412. Samples were included if they met these criteria: (1) WHO grade IV GBM; (2) gene expression profiles and survival information available; and (3) no preoperative radiotherapy or chemotherapy. Ultimately, 1097 out of the 1630 samples were included. A summary of all samples is detailed in **Supplementary Table 1**. All cohorts with complete OS data were used to construct and validate the PMLS. In addition, TCGA-GBM and GSE74187, which have PFS information, were also applied to appraise the model’s performance in predicting progression. For TCGA-GBM, the FPKM normalized data from the TCGA GDC portal were modified to transcripts per kilobase million (TPM) followed by log-2 transformation. Both mRNA and lncRNA were extracted pursuant to the gene annotations in GENCODE (Homo sapiens GRCh38), and the corresponding ENSEMBLES IDs were named the row names of all expression matrices for subsequent analysis.

### Signature generation through integrated machine learning algorithms

Following the methods previously reported in elegant studies (14, 15), gene expression profiles from all cohorts were transformed into z score to enhance the comparability among different cohorts. The following pipeline was executed to develop the PMLS model based on the 10 cohorts (TCGA-GBM, c301, c325, GSE13041, GSE42669, GSE43378, GSE74187, GSE83300, GSE4271, and GSE4412) with complete expression and survival information:

1. Univariate Cox regression analysis was performed separately in each of the ten cohorts to screen genes with reproducible survival associations. Because several validation cohorts had relatively small sample sizes and were generated on different transcriptomic platforms, a stringent cohort-specific multiple-testing threshold could have excluded genes with moderate but reproducible prognostic effects. Therefore, we used an unadjusted P < 0.1 as a discovery-level threshold rather than a definitive significance criterion. To reduce the inclusion of cohort-specific or directionally inconsistent signals, candidate genes were required to show the same HR direction and meet the P < 0.1 threshold in more than six of the ten cohorts. This criterion was designed to prioritize cross-cohort stability and directional consistency before model construction. The resulting SSRGs were then subjected to the integrated machine-learning framework, and the final model was selected according to its average C-index across independent validation cohorts, providing an additional safeguard against overfitting and cohort-specific false-positive findings.
2. GSE13041 was employed to develop the PMLS model for possessing the largest sample size. Ten machine learning algorithms including CoxBoost, elastic network (Enet), generalized boosted regression modeling, Lasso, partial least squares regression for Cox (plsRcox), random survival forest (RSF), Ridge, stepwise Cox, supervised principal components (SuperPC), and survival support vector machine (survival-SVM), were integrated to construct the PMLS with outstanding accuracy and robustness. The algorithms that possessed feature selection capability namely CoxBoost, Lasso, RSF, and stepwise Cox were amalgamated to generate a consensus model. A total of 76 algorithm combinations were conducted on SSRGs to fit prognostic models by virtue of 10-fold cross-validation. The detailed descriptions are generalized in the **Supplementary Material**.
3. All models originating from the 76 algorithm combinations were further examined in the remaining 9 datasets. For each model, its C-indices across all validation cohorts were calculated, and the model harboring the highest average C-index was considered the optimum one.

### Published signatures collection

To examine whether our selective model has superior performance over other signatures previously proposed, we recalled signatures in glioma from PubMed (https://pubmed.ncbi.nlm.nih.gov/) as comprehensively as possible, excluding miRNA signatures due to the extreme lack of miRNA expression data. These signatures were fitted by various machine-learning approaches, including Lasso, RSF, and stepwise Cox, and derived from a variety of biological processes, such as heme biosynthesis, glycolysis, fatty acid metabolism, iron metabolism, inflammation, autophagy, ferroptosis, pyroptosis, necrosis, hypoxia, oxidative stress, RNA binding protein, endoplasmic reticulum stress, and epithelial-mesenchymal transition (**Supplementary Table 2**). According to the signatures, the C-indices were calculated following the univariate Cox regression analysis carried out across all cohorts.

### Investigation of biological function

Gene set enrichment analysis (GSEA) was carried out to explore the latent biological processes and mechanisms underlying PMLS. Enriched terms regarding hallmarks, Kyoto Encyclopedia of Genes and Genomes (KEGG) pathways, and biological processes of gene ontology (GO) were identified. The input gene set files were procured from the MSigDB online webtool (version 7.5.1, h.all.v7.5.1.symbols.gmt, c2.cp.kegg.v7.5.1.symbols.gmt, and c5.go.v7.5.1.symbols.gmt) while the input genes were a list of mRNAs sorted in descending order of their correlation coefficients with PMLS scores which were calculated by means of Pearson’s correlation test. The *clusterProfiler* package was employed to perform GSEA based on the ranked gene list. Gene sets with |normalized enrichment score (NES)| >1 and false discovery rate (FDR) <0.05 were deemed significantly enriched. The Progeny algorithm inferred the relative activity of the upstream signaling pathway by detecting the expression characteristics of a small number of stable downstream target genes.

### Characterizing immune-related features

The expression profiles of immune modulators were summarized across co-stimulatory molecules, co-inhibitory molecules, ligands, receptors, cell-adhesion molecules, antigen-presentation molecules, and other immune-related markers. To characterize the immune microenvironment associated with PMLS, multiple deconvolution and immune-estimation algorithms were applied to the transcriptomic profiles, including CIBERSORT, CIBERSORT-ABS, MCPcounter, xCell, EPIC, ESTIMATE, TIMER, quanTIseq, and IPS. TIDE-related immune evasion scores, cytolytic activity scores (CYT), and submap algorithms were applied to infer potential responses to immunotherapy.

### Pan-cancer analysis

Given its potential importance within the PMLS and has not been well characterized in GBM, *LPGAT1* was selected for further investigation. We analyzed pan-cancer mutation, copy number variation (CNV), and DNA methylation data from TCGA to investigate the potential mechanisms by which *LPGAT1* dysregulation may drive tumorigenesis. The associations between *LPGAT1* expression and survival outcomes, clinicopathological features, and biological pathway activity were also assessed.

### Immunofluorescence

Human glioma tissue microarrays (HBraG180Su01) were purchased from Shanghai Outdo Biotech Co., LTD (Shanghai, China) for immunofluorescence analysis of *LPGAT1* expression. Formalin-fixed, paraffin-embedded sections were deparaffinized in xylene, rehydrated through graded ethanol, and subjected to heat-mediated antigen retrieval. After permeabilization and blocking with 5% BSA, sections were incubated with primary antibodies against *LPGAT1* (Abcam, ab230647, 1:500) and tumor-associated marker IL-13Rα1 (Abcam, ab79277, 1:500) at 4°C overnight. The sections were then washed and incubated with Alexa Fluor 488 and Alexa Fluor 594-conjugated secondary antibodies (Thermo Fisher scientific, A-11008, A-11012, 2μg/ml) for 1 hour at room temperature, followed by nuclear counterstaining with DAPI (Thermo Fisher scientific, 62248, 1μg/ml) for 15 minutes. Images were captured using a fluorescence scanning instrument (KFBIO, KF-PRO). Quantification was performed by measuring the mean fluorescence intensity of *LPGAT1* in defined regions of interest, with background subtracted from each image.

### RT-qPCR assays

Total RNA was extracted from normal human astrocyte controls (NHA and HA1000) and glioma cell lines (LN18, U87, U118, A172, LN229, U251, and SF295) using RNA extraction reagent (Yeasen, 19211ES60) following the manufacturer’s instructions. RNA concentration and purity were determined using Nanodrop (ThermoFisher, NanoDrop One). For each sample, 1μg of total RNA was reverse-transcribed into cDNA using reverse transcription kit (Yeasen, 11151ES60). RT-qPCR was performed using SYBR Green qPCR Master Mix (Yeasen, 11201ES08) on a real-time PCR system (Long Gene, Q2000C). *GAPDH* served as the internal control, and relative *LPGAT1* mRNA expression was calculated using the 2^-ΔΔCt^ method, also known as the Livak method (16), with GAPDH as the endogenous reference gene. Briefly, ΔCt was calculated as Ct (*LPGAT1*) - Ct (*GAPDH*), and ΔΔCt was calculated relative to the indicated control group. Relative expression was then determined as 2^-ΔΔCt^. All reactions were run in technical triplicates. Primer sequences used for RT-qPCR are provided in **Supplementary Table 4**.

### Promising therapeutic agent exploration employing cancer cell line data

Expression profiles of human cancer cell lines (CCLs) were acquired from the Broad Institute Cancer Cell Line Encyclopedia (CCLE) project. Drug sensitivity information of CCLs was retrieved from the Cancer Therapeutics Response Portal (CTRP) and the PRISM Repurposing dataset which incorporated the sensitivity data of 481 compounds of 835 CCLs and 1448 compounds of 482 CCLs respectively. The area under the dose-response curve (AUC) value was used as a metric to evaluate drug sensitivity, which increased with the decrease in AUC values.

### Statistics

All data processing, statistical analysis, and plotting were performed in R 4.3.2 software. Pearson’s correlation coefficients were utilized to compare the correlations between continuous variables. The t-test or Wilcoxon rank-sum test was conducted to compare the differences between two groups. All statistical tests were two-sided, and P < 0.05 was considered statistically significant.

## Results

### Integrative development of a consensus signature

**Figure 1:**
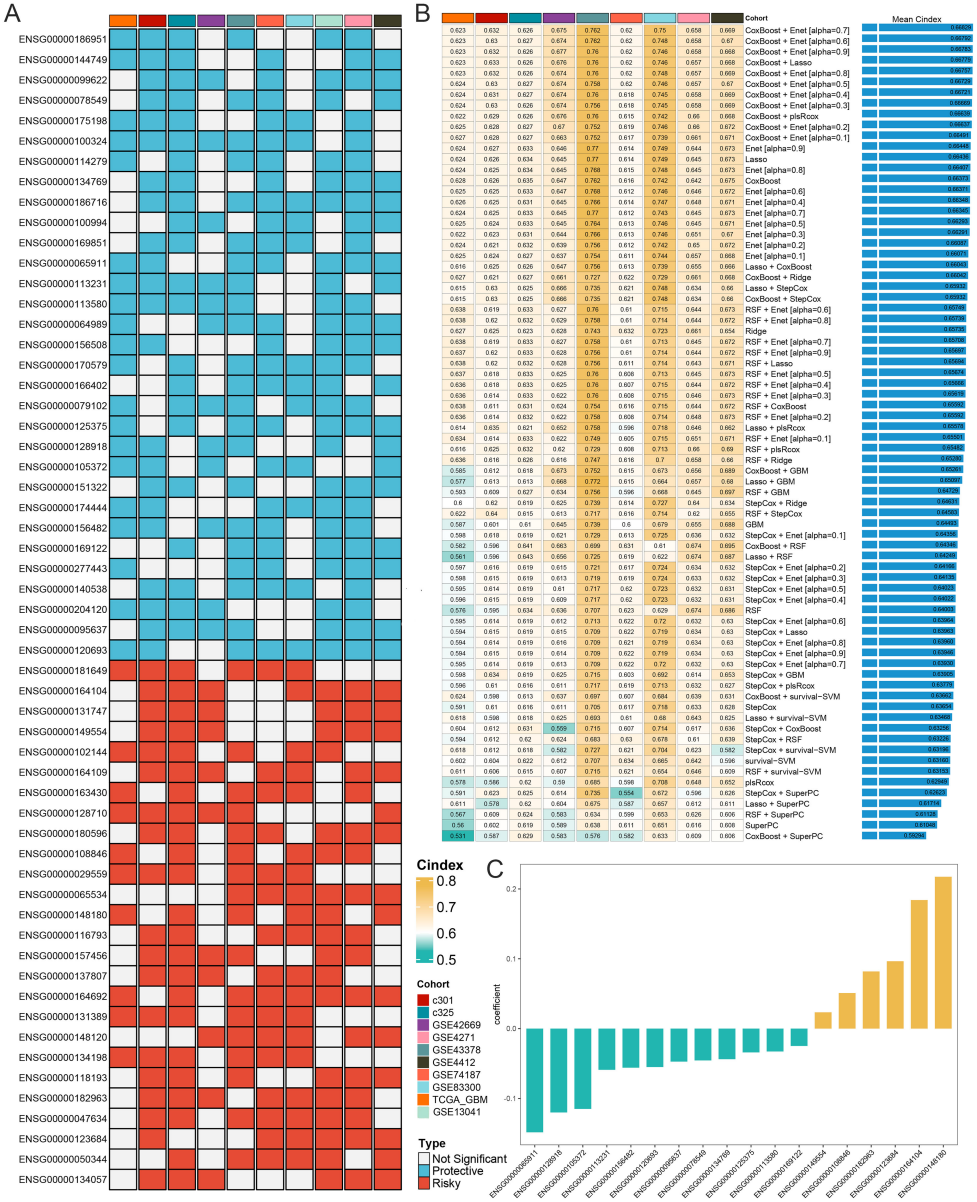
Integrative construction of the purificatory machine learning-derived gene signature. **(A)** 57 stable survival-related genes across 10 cohorts based on univariate Cox regression analysis. **(B)** The C-indexes of 76 kinds of prediction models in nine validation cohorts. **(C)** Coefficients of 18 genes finally obtained through CoxBoost followed by Enet (α=0.7).

Based on all ten cohorts, a total of 57 SSRGs with stable prognostic significance were recognized by performing univariate Cox regression analysis and further subjected to the integrated machine learning framework to fit a consensus model (**Figure 1A**). In the GSE13041 dataset, the C-indices were calculated to measure the performance of prediction models fitted by 76 kinds of machine learning combinations through the 10-fold cross-validation framework. In the training dataset, the C-indices reached extremely high levels in a few algorithms, even exceeding 0.9, such as RSF. To minimize the reduction of model reliability due to overfitting effects and test whether the model still retains robust predictive performance across different validation datasets, which is called its generalization capability, the average C-indices were calculated in the 9 other validation cohorts, and the highest one was considered the best. The combination of CoxBoost and Enet (alpha = 0.7) demonstrated the largest average C-index (0.668) and was selected as the optimal model for subsequent analysis (**Figure 1B**).

The CoxBoost model was finally set via the *CoxBoost* function after determining the optimal penalty via the LOOCV routine *optimCoxBoostPenalty* function and selecting the number of boosting steps via the cv.*CoxBoost* function. Subsequently, Enet was implemented via the *glmnet* package with the regularization parameter determined through 10-fold cross-validation and the L1-L2 trade-off parameter set to 0.7, leading to a final set of 18 SSRGs, including *ENSG00000078549, ENSG00000134769, ENSG00000065911, ENSG00000113231, ENSG00000113580, ENSG00000125375, ENSG00000128918, ENSG00000105372, ENSG00000156482, ENSG00000169122, ENSG00000095637, ENSG00000120693, ENSG00000164104, ENSG00000149554, ENSG00000108846, ENSG00000148180, ENSG00000182963*, and *ENSG00000123684*. For each patient, a risk score was calculated based on the expression levels of 18 SSRGs weighted by the corresponding regression coefficients (**Figure 1C**). The 18 SSRGs with their annotation information are listed in **Supplementary Table 3**.

### Independent prognostic value of PMLS

**Figure 2:**
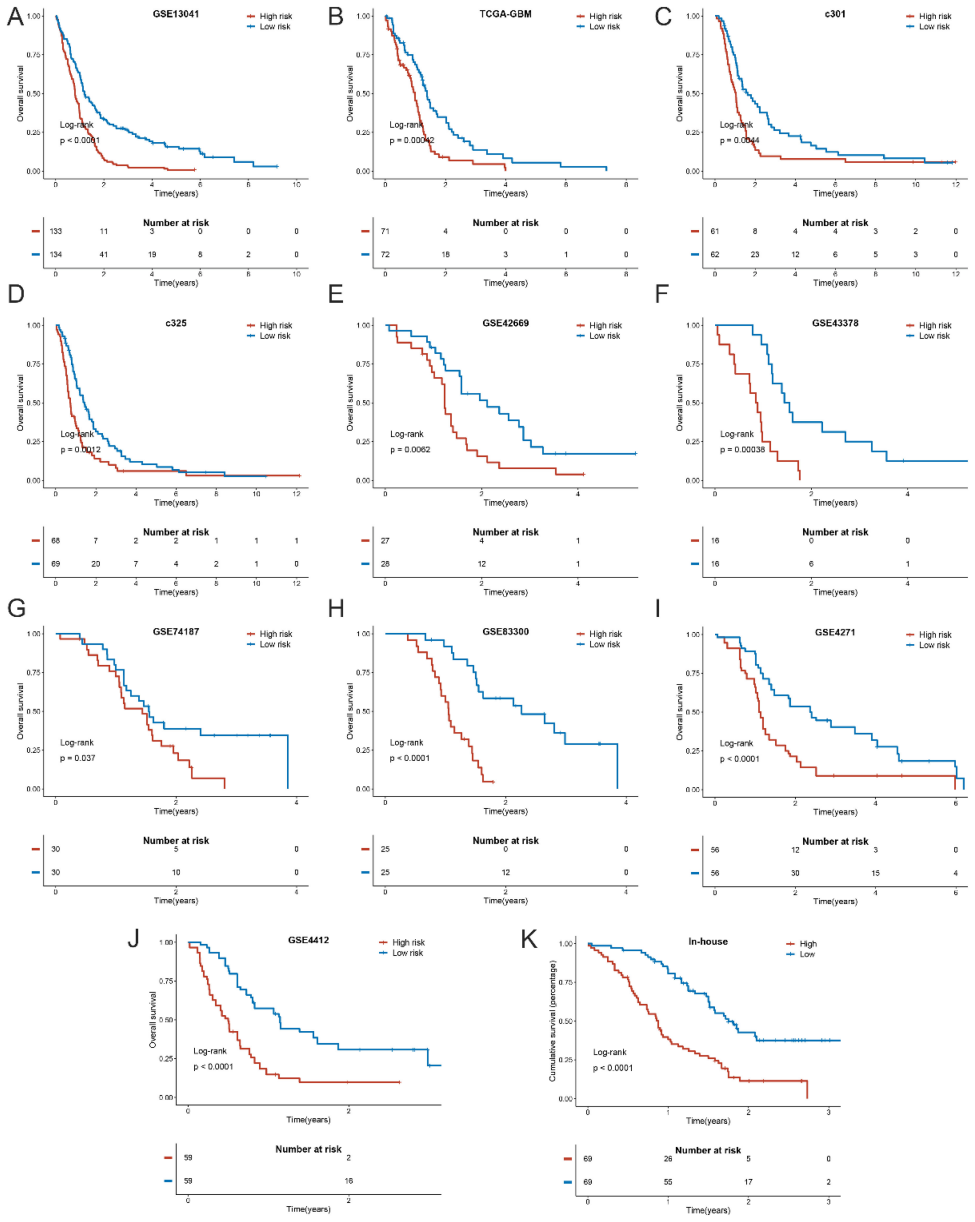
Kaplan-Meier survival analysis of PMLS. **(A–J)** Kaplan-Meier curves of OS according to the PMLS in GSE13041 **(A)**, TCGA-GBM **(B)**, c301 **(C)**, c325 **(D)**, GSE42669 **(E)**, GSE43378 **(F)**, GSE74187 **(G)**, GSE83300 **(H)**, GSE4271 **(I)**, and GSE4412 **(J)**.

The median risk score bisected the patients into high- and low-risk groups. Kaplan-Meier analysis demonstrated that high-risk patients lived for markedly shorter times compared to the low-risk group in the training dataset GSE13041 (n = 267, log-rank test: P < 0.0001), and similar results were achieved in 9 validation datasets TCGA-GBM (n = 143, log-rank test: P = 0.00042), c301 (n = 123, log-rank test: P = 0.0044), c325 (n = 137, log-rank test: P = 0.0012), GSE42669 (n = 55, log-rank test: P = 0.0062), GSE43378 (n = 32, log-rank test: P = 0.00038), GSE74187 (n = 60, log-rank test: P = 0.037), GSE83300 (n = 50, log-rank test: P < 0.0001), GSE4271 (n = 112, log-rank test: P < 0.0001), and GSE4412 (n = 118, log-rank test: P < 0.0001) (**Figures 2A–J**). We also retrieved disease-specific survival (DSS) data from the TCGA-GBM dataset and progression-free survival (PFS) data from both the TCGA-GBM dataset and GSE74187. As expected, patients in the high-risk group exhibited conspicuously shorter DSS and PFS (**Supplementary Figures 2A–C**).

Multivariate Cox regression analysis was applied to evaluate whether the prognostic significance of the PMLS model was independent of clinical traits and molecular features. GSE74187 was extracted due to a lack of clinical information. In the other 9 datasets, our PMLS was still statistically significant for OS even when adjusted for age, gender, PRS_type, chemotherapy, radiotherapy, temozolomide, TCGA_subtypes, Karnofsky performance status (KPS), IDH, MGMTp, and 1p19q state mirroring its capacity for assuming the role of an independent risk factor for GBM patients (**Supplementary Figures 2D–L**).

The status of IDH and MGMTp has a profound and considerable impact on the prognosis and even the options of treatment modalities of GBM (17). Patients harboring IDH mutation and MGMTp methylation have shown better outcomes (18). We therefore investigated the relevance of these two molecular features for the PMLS. As expected, the IDH mutation group and the MGMTp methylation group showed a tendency toward lower PMLS scores in both c301 and c325 (**Supplementary Figures 2M–P**).

### Evaluation of the PMLS model

**Figure 3:**
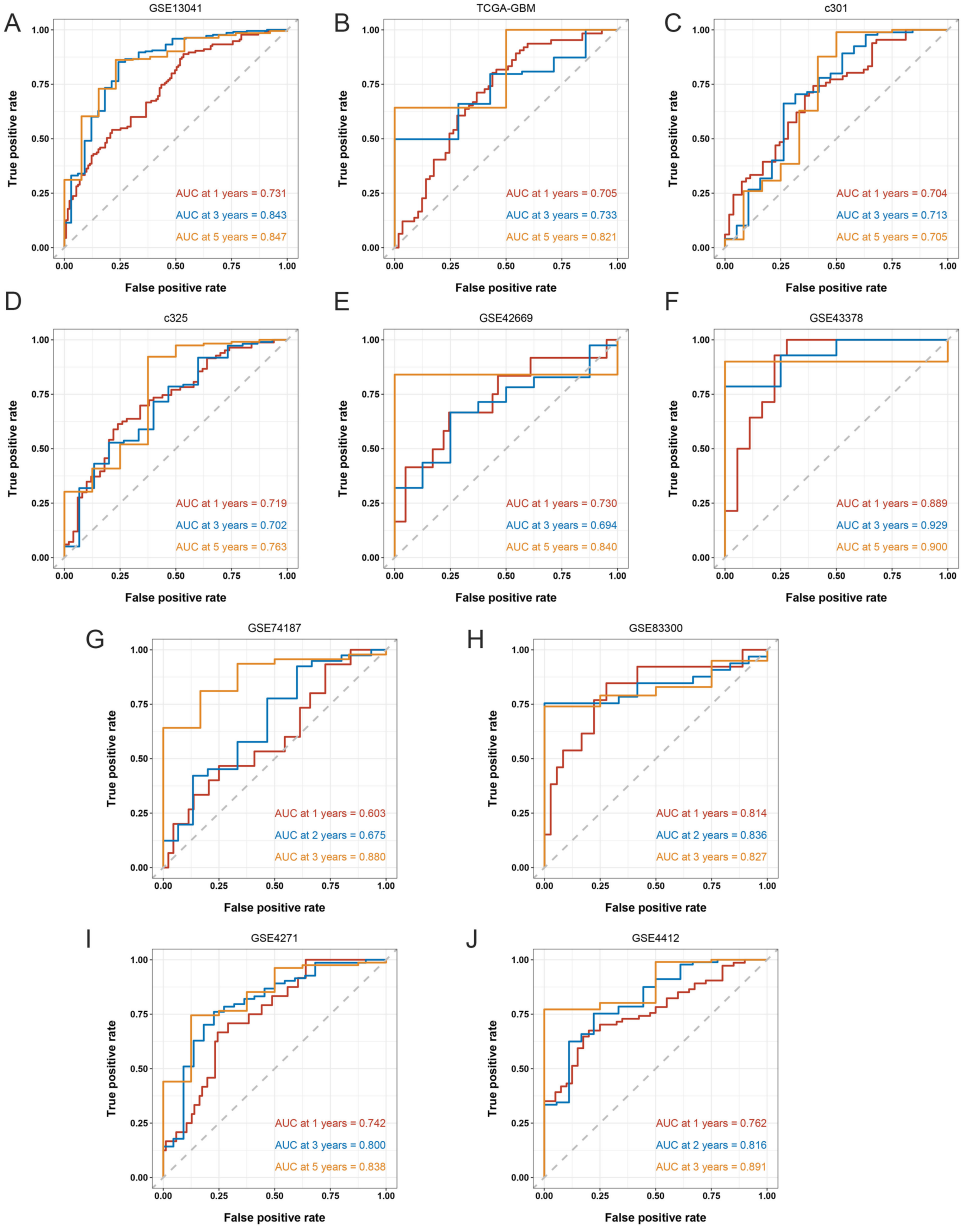
Time-dependent ROC analysis for predicting OS. **(A–J)** ROC curves of OS according to the PMLS in GSE13041 **(A)**, TCGA-GBM **(B)**, c301 **(C)**, c325 **(D)**, GSE42669 **(E)**, GSE43378 **(F)**, GSE74187 **(G)**, GSE83300 **(H)**, GSE4271 **(I)**, and GSE4412 **(J)**.

**Figure 4:**
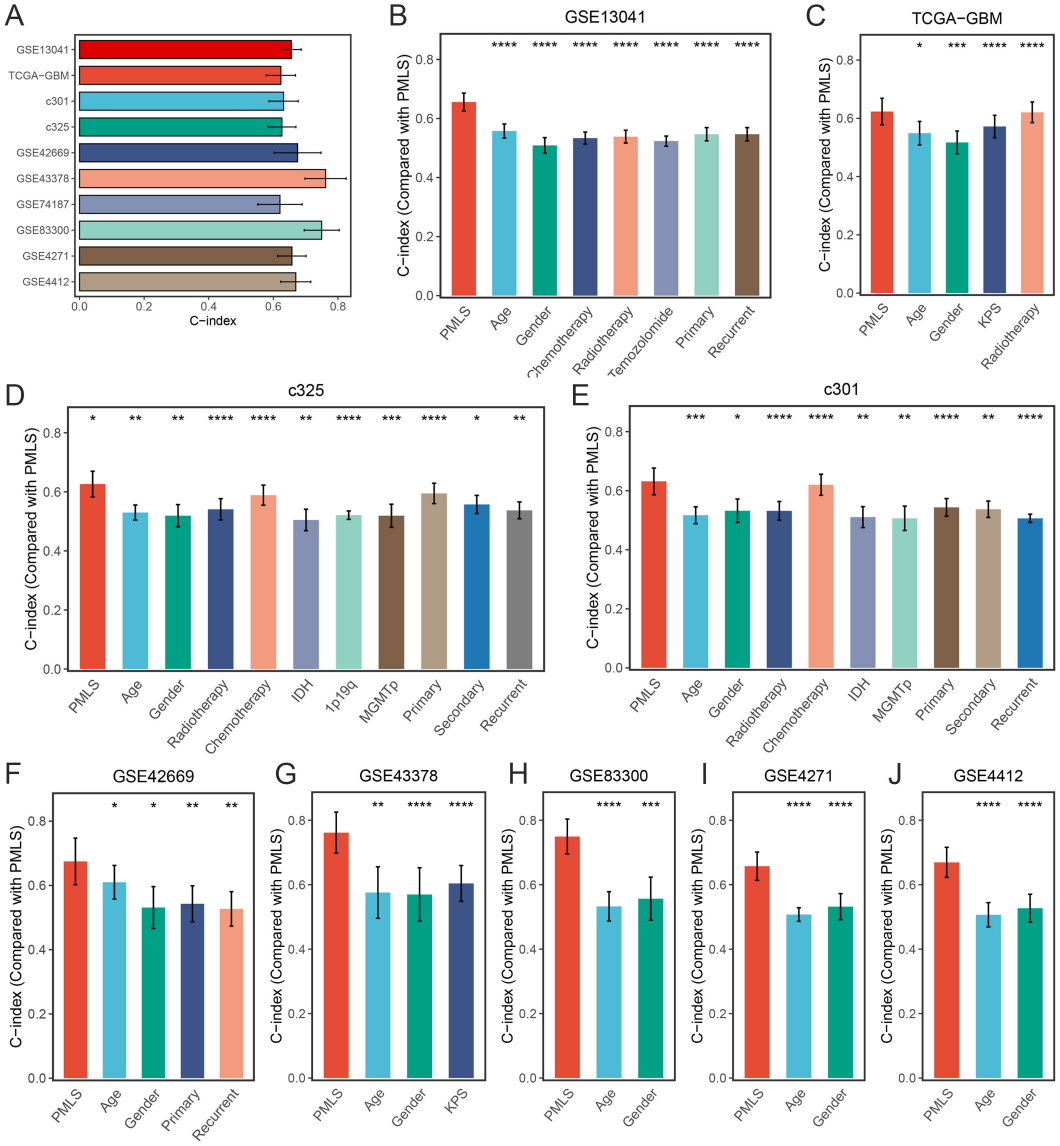
Robust performance of the PMLS. **(A)** The C-indexes of PMLS across all cohorts. **(B–J)** The performance of the PMLS was compared with common clinical and molecular characteristics in predicting prognosis in all training and validation datasets. Z-score test: *P < 0.05, **P < 0.01, ***P < 0.001, ****P < 0.0001.

The timeROC package was utilized to assess the predictive efficacy of our PMLS model. Almost all AUCs exceeded 0.7 at 1/3/5 (or 1/2/3) years in the following cohorts, GSE13041 (0.731/0.843/0.847), TCGA-GBM (0.705/0.733/0.821), c301 (0.704/0.713/0.705), c325 (0.719/0.702/0.763), GSE83300 (0.814/0.836/0.827), GSE4271 (0.742/0.800/0.838), and GSE4412 (0.762/0.816/0.891), or even reached 0.9 in GSE43378 (0.889/0.929/0.900). Only 3 AUCs were below 0.7, in GSE42669 (0.730/0.694/0.840) and GSE74187 (0.603/0.675/0.880) (**Figures 3A–J**). In the 10 cohorts, the C-indices [95% confidence interval] were 0.656 [0.636-0.676], 0.623 [0.593-0.654], 0.632 [0.601-0.662], 0.626 [0.597-0.655], 0.675 [0.627-0.723], 0.762 [0.719-0.804], 0.620 [0.575-0.666], 0.750 [0.713-0.786], 0.658 [0.628-0.687], and 0.669 [0.638-0.701], respectively (**Figure 4A**). Overall, the above results demonstrated the favorable and robust performance of our PMLS model for prognostication.

Epigenetic and molecular tumor heterogeneity have been proven to be closely linked to patient survival in glioblastoma (19). We thus compared the superiority of PMLS with available clinical and molecular variables for predicting outcomes of GBM. In 10 cohorts, PMLS was observed to show significantly superior utility over all clinical characteristics, including age, sex, PRS_type, chemotherapy, radiotherapy, temozolomide, TCGA_subtypes, KPS, IDH, MGMTp, and 1p19q state (**Figures 4B–J**).

### Comparisons of published prognostic signatures

**Figure 5:**
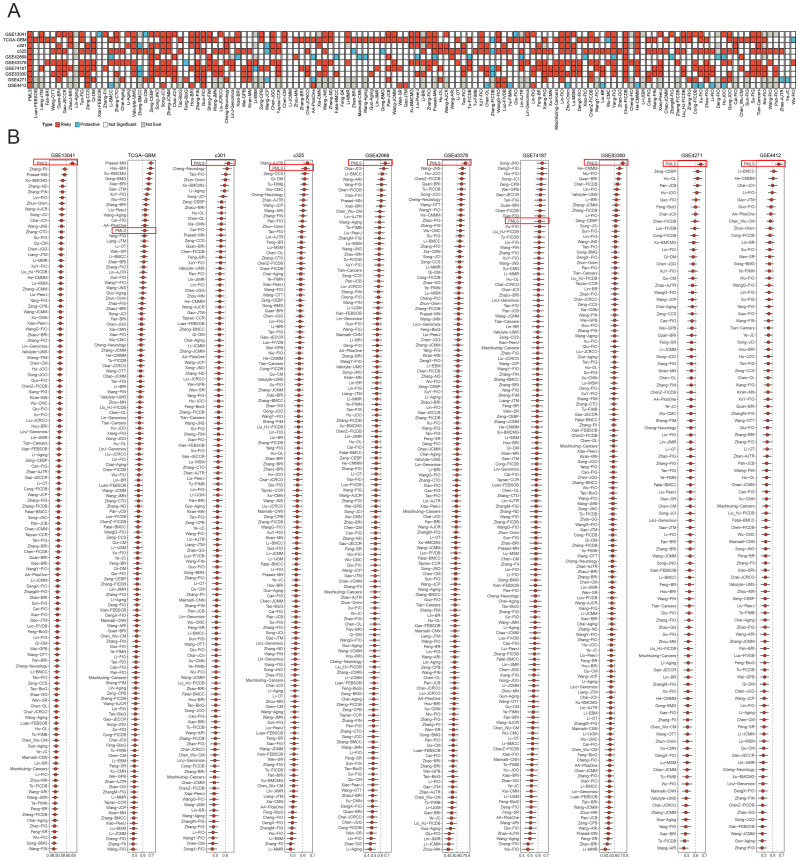
Comparisons of prognostic gene expression signatures. **(A)** Univariate Cox regression analysis of the PMLS and 135 published signatures. **(B)** The C-indexes of PMLS and 135 published signatures in GSE13041, TCGA-GBM, c301, c325, GSE42669, GSE43378, GSE74187, GSE83300, GSE4271, and GSE4412.

Dramatically increasing numbers of predictive gene signatures have been proposed as high-throughput sequencing technologies and big-data processing capabilities evolve (20). To compare the power of PMLS with other signatures, we comprehensively recruited a total of 135 signatures comprising mRNAs and lncRNAs with miRNA signatures excluded owing to the serious lack of available miRNA expression data (**Supplementary Table 2**). For each signature, univariate Cox regression analysis was performed, and only our PMLS model retained consistent significance across all cohorts (**Figure 5A**). The C-indices were further calculated in all cohorts. As shown in **Figure 5B**, the predictive power of our model ranked first in seven of the ten cohorts, including GSE13041, c301, GSE42669, GSE43378, GSE83300, GSE4271, and GSE4412. The PMLS ranked only second to Wang-AJCR, which only performed well in its development cohort, whereas it was far behind the PMLS in other cohorts. Similarly, in the TCGA and GSE74187 cohorts, signatures that performed better than our PMLS were far worse in other cohorts. This may be due to a poor generalization ability caused by model overfitting and provides indirect proof that the PMLS boasted strong robustness and recapitulation feasibility. To further compare PMLS with robust competing models, we focused on published signatures that showed recurrent prognostic associations across more than eight cohorts. We summarized the cohort-specific C-index, mean C-index, and gene number of PMLS and five representative robust published signatures, including Zeng-CEBP, Song-JCI, Zhang-FIN, Lin-FIO, and Chen-FICDB (**Supplementary Table 2**). The C-index of PMLS was the highest among 9 out of 10 cohorts, and PMLS achieved the highest mean C-index across the ten cohorts (0.667), exceeding Chen-FICDB (0.585), Song-JCI (0.579), Zhang-FIN (0.56), Zeng-CEBP (0.571), and Lin-FIO (0.559). In addition, PMLS retained a moderate model size of 18 genes, compared with 4 genes in Zeng-CEBP, 20 genes in Song-JCI, 5 genes in Zhang-FIN, 5 genes in Lin-FIO, and 11 genes in Chen-FICDB, suggesting a favorable balance between cross-cohort predictive performance, model complexity, and potential clinical feasibility.

### Construction of a nomogram based on PMLS and clinical features

The above results greatly reinforced the belief that our PMLS may possess promising prospects and unprecedented potential in discerning high-risk patients with GBM. Nomograms are customized to the profile of an individual patient and widely applied for tumor prognosis, principally due to their capacity to reduce the statistical models into a single numerical estimate of the probability of an event, such as recurrence or death, contributing to informing clinical decision-making during clinical encounters by generating user-friendly graphical interfaces (21). The *RMS* package was used to compile the PMLS scores with the patients’ clinicopathological data and to construct a predictive nomogram. To avoid optimistic bias caused by using the model-training cohort, we constructed the nomogram in an independent validation cohort rather than in GSE13041. The CGGA c325 cohort was selected because it had a relatively large sample size among the validation cohorts and contained comparatively complete clinicopathological and molecular annotations required for nomogram construction, including age, gender, treatment information, IDH mutation status, MGMT promoter methylation status, 1p/19q codeletion status, and PRS type. Therefore, c325 was used to integrate PMLS with available clinical and molecular variables for nomogram development (**Supplementary Figure 3A**). It is inspiring to see in the ROC curves that the nomogram we constructed can quite accurately predict the survival of patients, with AUCs at 1/3/5 years of 0.804/0.780/0.837, which even outperformed the single PMLS (**Supplementary Figure 3B**). Furthermore, the calibration curves also indicated the excellent performance of the nomogram at 1/3/5 years (**Supplementary Figure 3C**).

### Underlying biological mechanisms of PMLS

**Figure 6:**
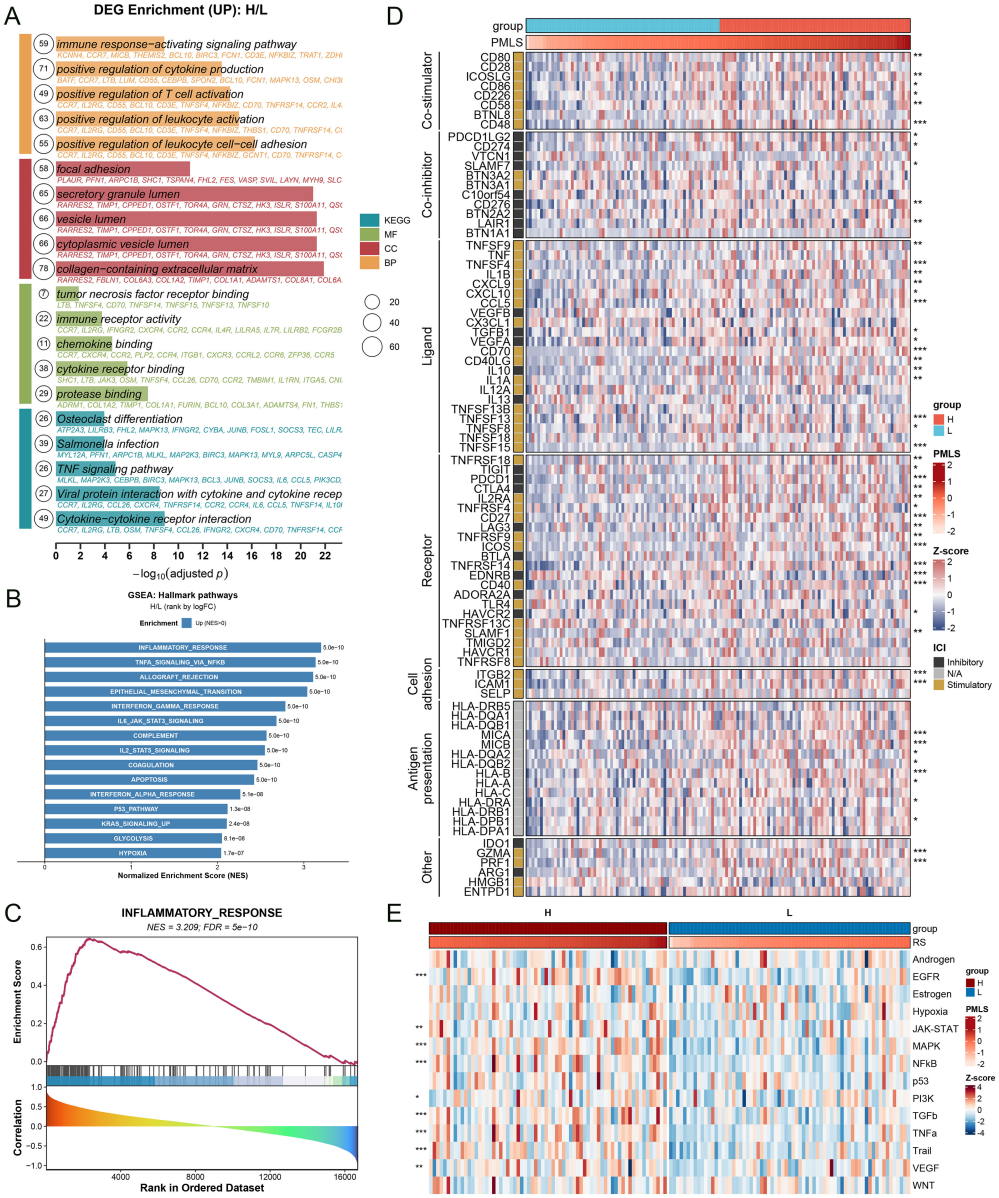
High PMLS scores are associated with inflammatory signaling and immunoregulatory molecules. **(A)** Enrichment analysis of genes upregulated in the high-PMLS group. **(B)** Hallmark GSEA showing pathways enriched in high-PMLS tumors. **(C)** GSEA curve for the inflammatory response hallmark signaling. **(D)** Heatmap of immunomodulatory molecules across co-stimulatory, co-inhibitory, ligand, receptor, cell-adhesion, antigen-presentation, and other immune-related features. **(E)** Heatmap of inferred pathway activity by progeny between high- and low-PMLS groups.

Scrutinizing the biological processes and molecular mechanisms underlying PMLS may have profound implications for deciphering the genesis and development of GBM especially for patients with high-risk scores. We first performed over-representation analysis (ORA) using genes upregulated in high-PMLS tumors to identify biological categories enriched among differentially expressed genes. To complement this threshold-dependent analysis, we then performed ranked GSEA using all genes ordered by their Pearson correlation coefficients with PMLS scores, which allowed us to evaluate pathway-level associations across the full transcriptome without relying on a differential-expression cutoff. Genes upregulated in the high-PMLS group were prominently enriched in immune response-activating signaling pathways, positive regulation of cytokine production, T-cell and leukocyte activation, leukocyte cell-cell adhesion, extracellular matrix-related terms, TNF receptor binding, immune receptor activity, chemokine binding, TNF signaling, viral protein interaction with cytokine and cytokine receptor, and cytokine-cytokine receptor interaction (**Figure 6A**). Hallmark GSEA further supported activation of inflammatory response, TNFA signaling via NFKB, allograft rejection, interferon responses, complement, IL6/JAK/STAT3 signaling, and cancer-related pathways like epithelial-mesenchymal transition, coagulation, apoptosis, KRAS signaling, glycolysis, and hypoxia were significantly enriched in high-PMLS tumors (**Figures 6B, C**). Consistently, the progeny algorithm inferred that the high-risk group was associated with oncogenic and immune-related pathways including EGFR, JAK-STAT, MAPK, NFKB, TGFb, TNFa, Trail, and VEGF (**Figure 6E**). Otherwise, the 15 most observably enriched pathways in each of the three gene sets were displayed in fgsea tables (**Supplementary Figures 4A–C**). The significantly enriched terms were mainly associated with the cell cycle, proliferation, immune response, and tumor-related pathways, providing clues to biological processes associated with aggressive GBM phenotypes. To provide a more comprehensive view of biological programs associated with PMLS, the pathways negatively associated with PMLS scores were exhibited in **Supplementary Figures 4D, E**. The results indicated that low-PMSL tumors may be related to synapse organization, CAM signaling, hedgehog_ signaling, etc.

### PMLS characterizes immune features and potential immunotherapy response

**Figure 7:**
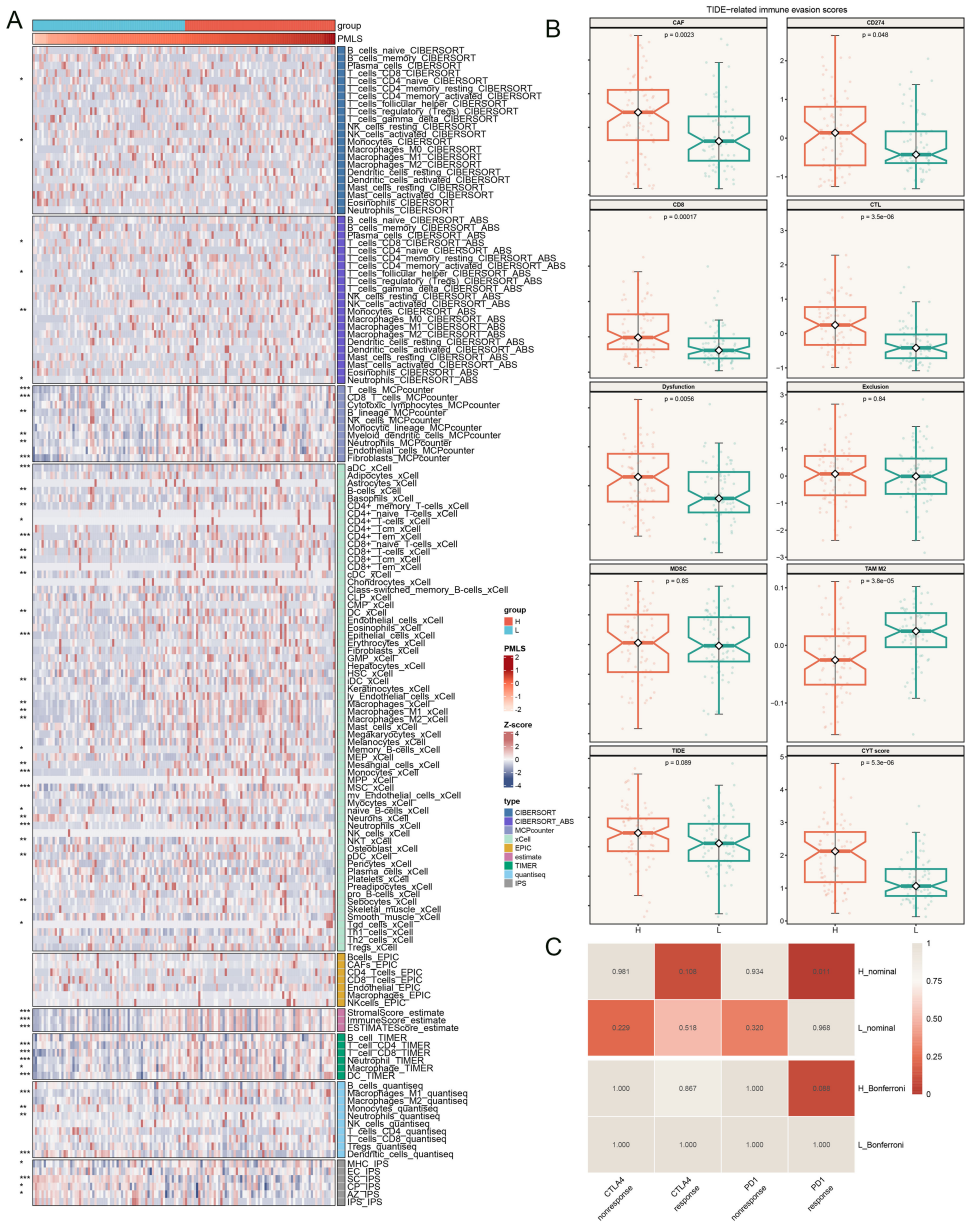
Immune infiltration and potential immunotherapy response characterization. **(A)** Integrated heatmap of immune-cell infiltration and immune/stromal features estimated using CIBERSORT, CIBERSORT-ABS, MCPcounter, xCell, EPIC, ESTIMATE, TIMER, quanTIseq, and IPS algorithms. **(B)** Comparison of TIDE-related scores and cytotoxic activity scores between high- and low-PMLS groups. **(C)** Predicted CTLA4 and PD1 response patterns.

Because immune-related pathways were strongly associated with PMLS, we examined whether the high-PMLS state was accompanied by broad immunoregulatory reshaping. Heatmap demonstrated that numerous co-stimulatory molecules, co-inhibitory molecules, ligands, receptors, cell-adhesion molecules, antigen-presentation molecules, and other immune-related genes were differentially distributed between PMLS groups and generally increased with risk scores in high-PMLS tumors (**Figure 6D**). In particular, high-PMLS tumors tended to show increased expression of representative immune checkpoint and immune regulatory genes, including *CD276*, *CD274*, *LAIR1*, *CD80*, *CD86*, *CTLA4*, *HAVCR2*, *LAG3*, and *TIGIT*, together with antigen-presentation and adhesion-related molecules such as *HLA* family genes and *ICAM1*. Several chemokine- or cytokine-related receptors and ligands, like *CCL5*, *CXCL10*, *IL10*, *CD40*, *CD27*, and *TLR4*, were also enriched in high-PMLS tumors, supporting an immune-inflamed but potentially immunosuppressive microenvironment (**Figure 6D**). For these pathway and immunoregulatory-marker analyses suggested that PMLS was closely associated with immune activation and immune-suppressive signaling, we next systematically evaluated the cellular composition of the tumor microenvironment and potential immunotherapy-related features using multiple independent computational approaches. Integrated heatmap analysis based on CIBERSORT, CIBERSORT-ABS, MCPcounter, xCell, EPIC, ESTIMATE, TIMER, quanTIseq, and IPS revealed extensive differences in cell abundance and immune or stromal scores between high- and low-PMLS groups (**Figure 7A**). Rather than being restricted to a single algorithm, several representative features showed consistent differences across methods. High-PMLS tumors tended to display higher myeloid-related signals, including macrophage/microglia-related signatures, neutrophils, and dendritic-cell features, together with increased stromal, endothelial, and immune-related scores. Some lymphoid features, including CD8 T-cell-related signals and cytolytic activity, were also higher in high-PMLS tumors, suggesting that immune activation may coexist with immunosuppressive myeloid and stromal components. In contrast, low-PMLS tumors generally showed weaker inflammatory and myeloid infiltration patterns. These results indicated a broad remodeling of the GBM tumor microenvironment across PMLS strata.

We further assessed the response to immunotherapy using TIDE, CYT, and submap algorithm. High-PMLS tumors showed significantly higher CD274, CD8, CTL, and cytolytic activity scores but less TAM M2, supporting enhanced immune activity, whereas upregulated CAF, dysfunction indicated that the immune activation may be accompanied by compensatory immunosuppressive mechanisms (**Figure 7B**). The submap results suggested that predicted response patterns differed between PMLS groups, with the PD1 response shown in the high-PMLS group (**Figure 7C**). These findings suggested that PMLS system may reflect immune activation and provide potential clues for immunotherapy sensitivity in GBM.

### *LPGAT1* drives tumor progression and affects prognosis

**Figure 8:**
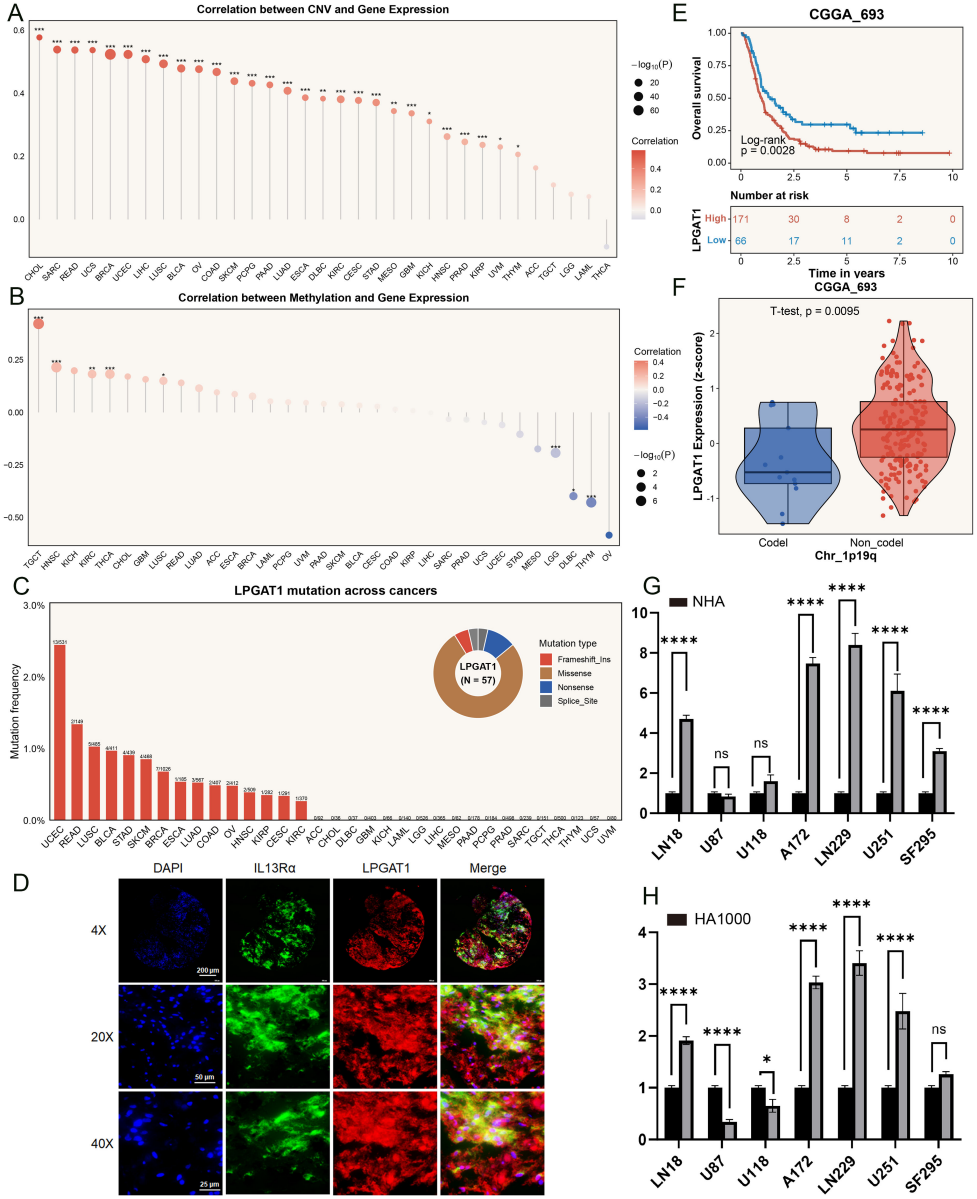
LPGAT1 is a tumor driver factor. **(A)** Correlation between LPGAT1 expression and copy number variation across cancers. **(B)** Correlation between LPGAT1 expression and methylation across cancers. **(C)** LPGAT1 mutation frequency and mutation types across cancers. **(D)** Tissue-level validation of LPGAT1 expression in glioma specimens. **(E)** Kaplan-Meier analysis of LPGAT1 expression. **(F)** LPGAT1 expression according to 1p/19q codeletion status. **(G, H)** Cell-line validation of LPGAT1 expression in glioma cells compared with controls.

Among the 18 genes retained in the final PMLS model, *LPGAT1* was selected for further investigation because of its relatively high model coefficient and limited characterization in GBM. Pan-cancer analysis showed that *LPGAT1* has a more extensive and stronger correlation with copy number variations compared to mutant phenotypes or DNA methylation. *LPGAT1* expression was positively correlated with CNV in majority of tumor types, suggesting that CNV may be an important mechanism contributing to *LPGAT1* dysregulation (**Figures 8A–C**). Mutation-frequency analysis indicated that *LPGAT1* mutations were present across several cancer types and were mainly composed of missense mutations, with additional frameshift, nonsense, and splice-site events (**Figure 8C**). Higher *LPGAT1* expression level was associated with worse overall survival and 1p/19q non-codeleted gliomas (**Figures 8E, F**), supporting a relationship between *LPGAT1* expression and more aggressive molecular phenotypes. Tissue staining and cell-line validation further supported dysregulated *LPGAT1* expression in glioma specimens and cell models compared with controls (**Figures 8D, G, H**).

Pan-cancer hallmarker correlation analyses showed that *LPGAT1* was extensively associated with malignant and inflammatory terms, including interferon responses, epithelial-mesenchymal transition, mitotic spindle, TNFA signaling via NFKB, G2M checkpoint, and KRAS signaling (**Supplementary Figure 5**). GO and KEGG enrichment highlighted the relationship between *LPGAT1* expression and nucleotide binding, intracellular organelle/cytoplasmic processes, mitochondrial components, protein modification, ubiquitin-mediated proteolysis, TNF signaling, metabolic pathways, platelet activation, T-cell receptor signaling, and other pathways (**Supplementary Figure 6**). These findings suggest that *LPGAT1* may serve as a potential biological target.

### Estimation of drug response regarding PMLS

**Figure 9:**
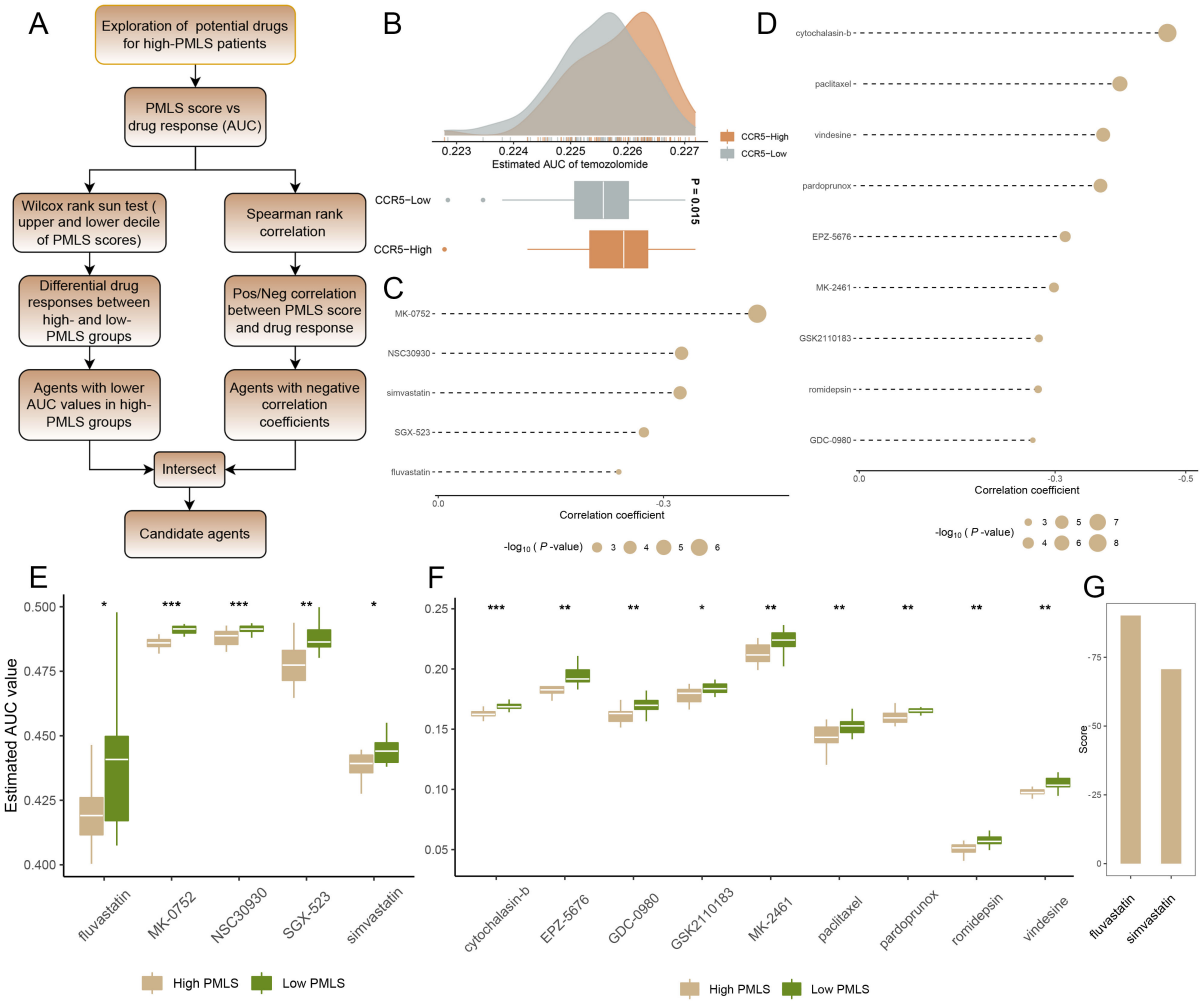
Identification of candidate agents. **(A)** Schematic strategies. **(B)** Comparison of estimated temozolomide’s sensitivity (logAUC) between high and low expression groups of CCR5. **(C, E)** The results of Spearman’s correlation analysis and differential drug response analysis of five CTRP-derived compounds. **(D, F)** The results of Spearman’s correlation analysis and differential drug response analysis of nine PRISM-derived compounds. Note that lower values on the y-axis of boxplots imply greater drug sensitivity. **(G)** Identification of promising therapeutic agents for high PMLS score patients according to the evidence from CMap analysis.

The CTRP and PRISM databases contain gene expression and drug sensitivity profiles of CCLs that can be used to construct predictive models of drug responses. The theoretical framework for screening potential drugs is visualized in **Figure 9A**. After cell lines originating from lymphoid and hematopoietic tissues and compounds with NAs existing in over 20% of all samples were excluded, the AUC value of each agent for each sample was estimated through the ridge regression model built into the pRRophetic package based on the expression data. Larger AUC values typified lower drug susceptibility. Before taking this further, we combined evidence from the previous literature with our estimation algorithm to demonstrate that the results of the drug sensitivity prediction were reliable. A previous study showed that C-C motif chemokine ligand 5 (*CCL5*) secreted by pericytes on GBM cells energizes C-C motif chemokine receptor 5 (*CCR5*) to enable DNA-dependent protein kinase catalytic subunit (DNA-PKcs)-mediated DNA damage repair (DDR) during temozolomide treatment and increased *CCL5-CCR5* paracrine signaling indicates a poor curative efficacy of TMZ (22). Hence, patients were dichotomized by the mid-value of their expression levels of *CCR5*, and the Wilcoxon run-sum test was utilized to compare the deviations of estimated AUC values of TMZ between differential expression clusters. The results demonstrated that patients with higher *CCR5* expression had significantly lower estimated AUC values (P = 0.015) (**Figure 9B**), conforming to how TMZ behaves clinically.

The drug response data derived from CTRP and PRISM were used to scrutinize candidate agents possessing higher drug sensitivity in high-risk group patients. First, agents with lower evaluated AUC values in the high-risk group were recognized through differential drug response analysis. Second, Spearman correlation analysis was carried out to identify drugs with negative correlations between AUC values and PMLS scores (r < -0.2 for CTRP or r < -0.3 for PRISM). Ultimately, the compounds obtained in the previous two steps intersected, resulting in 5 candidate drugs in CTRP (NK-0752, NSC30930, simvastatin, SGX-523, and fluvastatin) and 9 in PRISM (cytochalasin-b, paclitaxel, vindesine, pardoprunox, EPZ-5676, NK-2461, GSK2110183, romidepsin, and GDC-0980), with significantly higher sensitivity for high-risk GBM patients (**Figures 9C–F**). To increase the evidence supporting these candidate drugs as potential therapeutic strategies, CMap analysis was utilized to further screen compounds with posttreatment gene expression patterns opposite to the GBM-specific expression patterns. Then, the candidate drugs were intersected with those obtained in CTRP and PRISM, generating two compounds (simvastatin and fluvastatin) from CTRP (**Figure 9G**). Both candidate drugs have been shown to inhibit tumor cells and exhibit promising therapeutic efficacy for GBM patients (23, 24).

## Discussion

Glioblastoma exhibits inherent resistance to conventional treatments and almost inevitably relapses 6–9 months after initial diagnosis, regardless of treatments (25). In addition to TMZ, the FDA has approved four other drugs, but overall survival in GBM patients has not been prolonged in recent years (26). Due to its atypical early clinical symptoms and extreme aggressiveness, GBMs are often diagnosed at advanced stages (27). Hence, it is urgent to identify effective and robust biomarkers for patient screening, stratification, and treatment response to avoid over- or undertreatment as much as possible.

As whole genome sequencing technology and computational biology have become thriving and prosperous, emerging prognostic signatures have been proposed. The overwhelming majority of them originate from specific biological processes and pathways, resulting in the likelihood of pivotal biological mechanism omission. Moreover, numerous predictive signatures are only constructed based on mRNAs or lncRNAs, which may cause the loss of crucial information as well. Therefore, our study integrated all mRNAs and lncRNAs (miRNAs were eliminated due to the current lack of gene profiles), and 57 genes associated with prognosis were identified according to our established pipeline, contributing to the recognition of potential biomarkers.

For a long time, generations of researchers have made attempts to predict the outcome of GBM and have established a series of signatures. Nevertheless, these algorithms were generally selected based on personal preferences or bias, which may lead to nonobjective or inappropriate conclusions. Researchers are supposed to deliberate on why a specific algorithm is utilized and what the optimal solution is. Therefore, to generate a consensus signature, we constructed 76 algorithm combinations based on 10 machine learning algorithms to further reduce the dimensionality of variables. The algorithm combination with the largest mean C-index among all independent validation cohorts was chosen, i.e., CoxBoost followed by Enet, to develop the PMLS. The number of individuals, namely the sample size, is a noteworthy concern in the design of a research study because of its impact on statistical power (28). In this study, 1097 patients from 10 independent cohorts were used to develop and validate the PMLS and our model demonstrated efficient and robust performance across all cohorts.

In clinical decision-making and prognostic assessment, the available reference information is clinical traits and molecular characteristics. For instance, patients under the age of 70 with a KPS greater than 70 (capable of independent self-care) usually accept palliative concurrent chemoradiotherapy (29). Whether the gene encoding the metabolic enzyme isocitrate dehydrogenase (IDH) is mutated is an important genetic distinction in GBM. Patients harboring wild-type IDH show a more invasive status than IDH-mutated GBM patients (15 months versus 31 months of OS respectively) (30). In addition to IDH status, MGMT is another biomarker with significant prognostic value. Enhanced TMZ response and improved survival characterize MGMT promoter-methylated tumors with epigenetic silencing, and TMZ only has moderate effects in MGMT promoter unmethylated tumors (29, 31). We thus compared the performance of PMLS with available clinical and molecular characteristics for predicting the outcome of GBM. The results showed that in 10 cohorts, the PMLS not only demonstrated an independent risk factor when adjusted for variates such as age, sex, PRS_type, chemotherapy, radiotherapy, temozolomide, TCGA_subtypes, KPS, IDH, MGMTp, and 1p19q state, but also exhibited strikingly superior predictive capacity according to the calculated C-indices, providing a promising platform for risk stratification of GBM patients.

Another of our unprecedented works was to compare the predictive performance of the PMLS with almost all signatures (originating from mRNAs or lncRNAs) that have been proposed for GBM. In total, 135 published signatures were retrieved, which comprised combinations of genes of a wide variety of functional pathways; however, most of them have not been clinically translated or even subjected to rigorous validation. Univariate Cox regression analysis showed that none of these signatures preserved prognostic significance in all cohorts aside from our model. Furthermore, in most cohorts, the PMLS maintained absolute ascendancy over almost all other signatures in terms of predicting the outcome of GBM patients. Notice that most signatures performed well only in their own training cohort and poorly in other cohorts. This is probably due to overfitting caused by the lack of stringent inferential validation and the irrationality of algorithm selection which in turn precipitated poor generalizability of the models. The comparison with robust published signatures provides a practical perspective on the balance between predictive performance and model complexity. Although several published signatures showed recurrent prognostic associations across multiple cohorts, their cross-cohort C-index values were generally lower than those of PMLS. PMLS achieved the highest mean C-index among the compared models while maintaining an intermediate panel size of 18 genes. By comparison, smaller models such as Zeng-CEBP and Lin-FIO may be easier to implement but showed lower average predictive performance, whereas Song-JCI contained a comparable or slightly larger number of genes but still had a lower mean C-index. These findings suggest that PMLS may offer a favorable trade-off between robustness, predictive accuracy, and assay complexity. From a translational perspective, the 18-gene PMLS could potentially be implemented using targeted transcriptomic assays, such as RT-qPCR panels, NanoString-based profiling, or targeted RNA sequencing, rather than whole-transcriptome profiling. However, routine clinical applications require standardized RNA extraction, quality control, normalization procedures, prespecified risk-score cutoffs, prospective multicenter validation, and evaluation of cost-effectiveness.

We are particularly interested in exactly underlying biological processes and mechanisms lead to high-risk patients with GBM. Patients with a high PMLS score demonstrated an activated cell cycle, dynamic proliferation, enhanced immune response, and upregulated tumor-related pathways. This finding corroborates previous studies. Histologically, GBM cells present high rates of microvascular proliferation, cell division and central areas of necrosis. The investigation of *LPGAT1* provides a gene-level bridge between the PMLS model and biological interpretation. *LPGAT1* is a member of the final 18-gene panel, and its expression was associated with copy-number and methylation features, worse survival, 1p/19q non-codel phenotype. *LPGAT1* may be a promising candidate target for its interaction with malignant progression and immune-related signaling in glioma. Our studies offer auspicious resources for further exploration of the mechanisms underlying the extreme malignancy of GBM. In addition, our findings will expedite identification and elucidation of more complicated pathways to develop new drugs as well as more timely and precise molecular diagnosis of tumors.

Researchers are imbued with a desire to navigate the clinical way forward for individual patients. The PMLS, which has excellent prognostication and includes common clinical features, was used to construct a user-friendly graphical-interface nomogram, which was proven to outperform the PMLS, suggesting its prominent clinical application value. GBM tumors are highly heterogeneous among individuals, making it nearly impossible to find a one-size-fits-all treatment approach for GBM patients. Unfortunately, due to the immunosuppressive environment and immune escape caused by the blood-brain barrier (BBB), numerous emerging immunotherapies have achieved little success in GBM (32). Therefore, one of the major aims of our study was to help select treatment measures for specific populations, which is essential for maximizing therapeutic efficacy. Two agents (simvastatin and fluvastatin) with higher sensitivity were identified for high-PMLS score GBM patients in this study. Statins function to reduce lipid levels, are typically prescribed for cardiovascular diseases and have been discovered to display anticancer activity (33). Simvastatin and fluvastatin can suppress numerous tumors and reduce the specific mortality of patients by affecting the proliferation and migration of cancer cells, such as breast cancer (34, 35), lung cancer (36, 37), and prostate cancer (38, 39). Indeed, their roles in glioma have also been reported but are understudied; hence, further research is required to demonstrate their potential for clinical applications (40, 41).

Despite the promising clinical significance of PMLS in GBM, a few limitations should be recognized. First, all samples contained in our work were derived from retrospective cohorts; thus, further validation in a prospective study is necessary. Second, the latent connection between PMLS and certain variants may be underexplored owing to the inadequate clinical and molecular characteristics in public databases. Finally, the roles of most genes from the PMLS have yet to be elucidated via *in vivo* and *in vitro* studies.

## Conclusion

Overall, we comprehensively retrieved data from multicenter GBM patients and integrated 10 machine learning algorithms after selecting the best combination to develop and validate a consensus model (termed the PMLS) that could independently predict the prognosis of GBM patients. Furthermore, our PMLS model also surpassed common clinical and molecular features in its predictive accuracy, and it was superior to most of the 135 published prognostic signatures in all cohorts. Further analyses suggest that PMLS reflects inflammatory signaling, immune microenvironment remodeling, and tumor progression.

## Data Availability

The original contributions presented in the study are included in the article/**Supplementary Material**. Further inquiries can be directed to the corresponding authors.

## Supplementary material

Supplementary Figure 1 The workflow of this study.

Supplementary Figure 2 Predictive performance evaluation of PMLS. **(A, B)** Kaplan-Meier curves of DSS and PFS according to the PMLS in TCGA-GBM. **(C)** Kaplan-Meier curve of PFS according to the PMLS in GSE74187. **(D–L)** Multivariate Cox regression analysis in GSE13041 **(D)**, TCGA-GBM **(E)**, GSE42669 **(F)**, c301 **(G)**, c325 **(H)**, GSE43378 **(I)**, GSE83300 **(J)**, GSE4271 **(K)**, GSE4412 **(L)**. **(M–P)** Correlation between the PMLS and IDH mutation and MGMTp methylation status in c301 **(M, N)** and c325 **(O, P)**.

Supplementary Figure 3 Development and validation of the nomogram. **(A)** The prognostic nomogram for predicting the 1-, 3-, and 5-year survivals. **(B)** Time-dependent ROC analysis for predicting OS at 1, 3, and 5 years. **(C)** Calibration curves of the nomogram in terms of agreement between predicted and observed 1-, 3-, and 5-year outcomes.

Supplementary Figure 4 Underlying biological pathways and distinct microenvironment patterns related to PMLS. GO **(A)**, KEGG **(B)**, and cancer hallmark **(C)** of gene set enrichment analysis (GSEA) according to the PMLS. **(D, E)** Enrichment analysis of the low-PMLS group.

Supplementary Figure 5 Pan-cancer hallmark pathway associations of LPGAT1. **(A)** Bubble plot showing correlations between LPGAT1 expression and hallmark pathway activity across cancer types. **(B)** Representative correlations between LPGAT1 expression and pathway scores, including interferon alpha response, epithelial-mesenchymal transition, mitotic spindle, TNFA signaling via NFKB, G2M checkpoint, and KRAS signaling up.

Supplementary Figure 6 LPGAT1-related GO and KEGG enrichment analysis. **(A)** GO enrichment analysis of LPGAT1-associated genes. **(B)** KEGG enrichment analysis of LPGAT1-associated genes.
