# Supplementary material for: A multicohort machine-learning prognostic signature reveals inflammatory immune remodeling and statin sensitivity in glioblastoma
Source: Front Oncol. 2026 Aug 5;16:1912201. doi: 10.3389/fonc.2026.1912201 (PMC13485557; doi:10.3389/fonc.2026.1912201)
Supplement: Supplementary file 1 [file Table2.docx]

**Signature generated from** **machine learning** **integrative approaches**

10 machine learning algorithms including CoxBoost, elastic network (Enet), generalized boosted regression modeling (GBM), Lasso, partial least squares regression for Cox (plsRcox), random survival forest (RSF), Ridge, stepwise Cox, supervised principal components (SuperPC), and survival support vector machine (survival-SVM) was integrated to construct the purificatory machine learning-derived gene signature (PMLS) with high accuracy and stability performance. A few algorithms possessed the ability of feature selection, such as Lasso, stepwise Cox, CoxBoost, and RSF. Thus, we combined these algorithms to generate a consensus model. In total, 76 algorithm combinations were conducted on SRRLs to fit prediction models based on 10-fold cross-validation. The initial signature discovery was performed in GSE13041. The RSF model was implemented via the *randomForestSRC* package. RSF had two parameters *ntree* and *mtry*, where *ntree* represented the number of trees in the forest and *mtry* was the number of randomly selected variables for splitting at each node. We used a grid-search on ntree and mtry using 10-fold cross-validation. All the pairs of (*ntree*, *mtry*) were formed and the one with the best C-index value was identified as the optimized parameters. The Enet, Lasso, and Ridge were implemented via the *glmnet* package. The regularization parameter, lambda, was determined by 10-fold cross-validation, whereas the L1-L2 trade-off parameter, α, was set to 0-1 (interval = 0.1). The stepwise Cox model was implemented via s*urvival* package. A stepwise algorithm using the AIC (Akaike information criterion) was applied. The CoxBoost model was implemented via *CoxBoost* package, which is used to fit a Cox proportional hazards model by componentwise likelihood-based boosting. For the CoxBoost model, we used LOOCV routine *optimCoxBoostPenalty* function to first determine the optimal penalty (amount of shrinkage). Once this parameter was determined, the other tuning parameter of the algorithm, namely, the number of boosting steps to perform, was selected via the function *cv.CoxBoost*. The dimension of the selected multivariate Cox model was finally set by the principal routine CoxBoost. The plsRcox model was implemented via *plsRcox* package. The *cv.**plsRcox* function was used to determine the number of components requested, and the *plsRcox* function was applied to fit a partial least squares regression generalized linear model. The SuperPC model was implemented via *superpc* package, is a generalization of principal component analysis, which generates a linear combination of the features or variables of interest that capture the directions of largest variation in a dataset. The superpc.cv function used the10-fold cross validation to estimate the optimal feature threshold in supervised principal components. To avoid problems with fitting Cox models to small validation datasets, it uses the "pre-validation" approach. The GBM model was implemented via the *gbm* package. Using the10-fold cross validation, the *cv.gbm* function selected index for number trees with minimum cross-validation error. The *gbm* function was used to fit the generalized boosted regression model. The survival-SVM model was implemented via *survivalsvm* package. The regression approach takes censoring into account when formulating the inequality constraints of the support vector problem.
